# Experimental study on the engineering properties of expansive soil treated with Al_13_

**DOI:** 10.1038/s41598-020-70947-6

**Published:** 2020-08-18

**Authors:** Jianbo She, Zheng Lu, Yahui Duan, Hailin Yao, Liang Liu

**Affiliations:** 1 State Key Laboratory of Geomechanics and Geotechnical Engineering, Institute of Rock and Soil Mechanics, Chinese Academy of Sciences, Wuhan, 430071 People’s Republic of China; 2 University of Chinese Academy of Sciences, Beijing, 100049 People’s Republic of China; 3 Hubei Key Laboratory of Geo-Environmental Engineering, Wuhan, 430071 People’s Republic of China; 4 School of Urban Construction, Wuchang University of Technology, Wuhan, 430223 People’s Republic of China; 5 Central-South Architectural Design Institute Co., Ltd., Wuhan, 430071 People’s Republic of China

**Keywords:** Geochemistry, Natural hazards, Geochemistry

## Abstract

This paper investigates the stabilization of expansive soil with hydroxy-aluminium (Al_13_) with Al/soil ratios of 0.10, 0.14, 0.18, 0.22 and 0.26 mmol/g. A series of laboratory tests were conducted to study the effects of Al_13_ on the mechanical properties of expansive soil, including Atterberg limits, grain size distribution, swell percent, swell pressure and unconsolidated-undrained (UU) triaxial strength. The results revealed that Al_13_ reduced the plasticity index, clay content and swelling potential and enhanced the shear strength of expansive soil. The minerals and micro-structural changes in the soil samples were also determined by X-ray diffraction (XRD) and scanning electron microscopy (SEM), and the stabilized soil showed a remarkable flocculated-agglomerated structure characterized by the densification of particle associations. The consistency of the changes in mechanical properties and micro-morphology indicated that expansive soil can be effectively improved with the use of Al_13_. Additionally, the optimum dosage of polynuclear hydroxy-Al (PHA) solution for stabilizing expansive soil was 0.18 mmol/g under the given properties of expansive soil and PHA utilized in this study.

## Introduction

Expansive soil is very sensitive to changes in moisture content and thus shows repeated swell-shrink behaviour, which will seriously damage construction in expansive soil areas^1–3^. To mitigate or avoid this problem, various remedial techniques have been devised that are classified as physical, mechanical or chemical stabilization methods. Among these treatment methods, the chemical stabilization method has achieved remarkable effects in view of its efficiency, replicability and reliability. Conventional stabilizers, such as fly ash, cement, rice husk ash, lime, gypsum and magnesium chloride, are often used to mitigate excessive swelling and improve the mechanical properties of expansive soils^4–17^. Moreover, civil engineers have made great attempts to find different materials to enhance the engineering properties of expansive soil in recent decades.

In this study, hydroxy-aluminium with the stoichiometry [AlO_4_Al_12_(OH)_24_(H_2_O)_12_]^7+^ (Al_13_) as an Al species in a polynuclear hydroxy-Al (PHA) solution has been considered to be a desired intercalator and chemical stabilizer for swelling clay minerals^18–20^. This Al_13_ polymer is the primary component of intercalated Al adsorbed in the crystal layer to form thermally stable pillaring clay^21^. The basal spacing of montmorillonite crosslinked with Al_13_ remains essentially unchanged after heating to 220 °C, and after rewetting, it is confined to the stage of crystalline swelling^22^. Additionally, previous studies have found that Al_13_ can greatly reduce the swelling potential of montmorillonite and greatly improve its physio-mechanical properties^19,23–25^.

Expansive soil is a particular clay soil that usually contains a dominant proportion of montmorillonite minerals, and its swelling and shrinking characteristics are primarily controlled by the properties of montmorillonite. Thus, Al_13_ has the potential to decrease the swelling potential and enhance the engineering properties of expansive soil^19,26^. However, there are few significant studies on stabilized expansive soil with Al_13_. Liu et al.^26^ indicated that Al_13_ can greatly reduce the free swelling ratio of expansive soil. However, the physio-mechanical properties and micro-morphological characteristics of expansive soil stabilized with Al_13_ have not been examined and remain unknown until now.

In this work, the influence of PHA solution on the physio-mechanical properties and micro-morphology of expansive soil were investigated. The physical changes of stabilized and natural samples were studied by determination of Atterberg limits and grain size distribution analyses, while swell percent and swell pressure tests were also performed. In addition, unconsolidated-undrained (UU) triaxial tests were used to assess the improvement in the strength of the stabilized expansive soil. Finally, the minerals and micro-structural changes were obtained, and the results were assessed by X-ray diffraction (XRD) and scanning electron microscopy (SEM). The results showed that Al_13_ can significantly enhance the engineering properties of expansive soil. Moreover, the optimum dosage of PHA solution was proposed under comprehensive consideration of the changes in the physio-mechanical properties.

## Materials and methods

### Materials

Al_13_ is a hydroxy-Al polymer with a hydrated radius of 9.5 Å, whose content in PHA solutions depends on the total Al concentration, degree of basification (OH/Al molar ratio), reaction temperature, alkali injection rate, ageing time and temperature^19,27–30^. The PHA solution can be obtained by the alkali neutralization titration method^31^. According to the synthetic method reported by Yao et al.^19^, the single factor method was used to determine the optimum ranges of operating conditions, and then orthogonal experimental design was adopted to summarize the optimum parameters for preparing the PHA solution with a relatively high content of Al_13_. According to the range analysis, the OH/Al molar ratio is the key factor, followed by the total Al concentration, but the effect of reaction temperature and alkali neutralization rate may be neglected. The results showed that the content of Al_13_ in the PHA solution increased with the OH/Al molar ratio and reached a maximum when the OH/Al molar ratio was 2.5. Furthermore, it was found that the effect of ageing time on the content of Al_13_ was weak. Therefore, the total Al concentration and OH/Al molar ratio should be strictly controlled when adopting the above method, and other parameters can be adjusted according to the actual situation. In this paper, the ageing temperature was controlled at room temperature (20 °C), which relatively conformed to the actual ageing conditions of the PHA solution, and the ageing time was set to 1 week. In addition, the total Al concentration was fixed at 0.1 mol/L, and the OH/Al molar ratio was controlled at 2.5 with a reaction temperature of 60 °C and an alkali neutralization rate of 50 mL/min.

**Figure 1:**
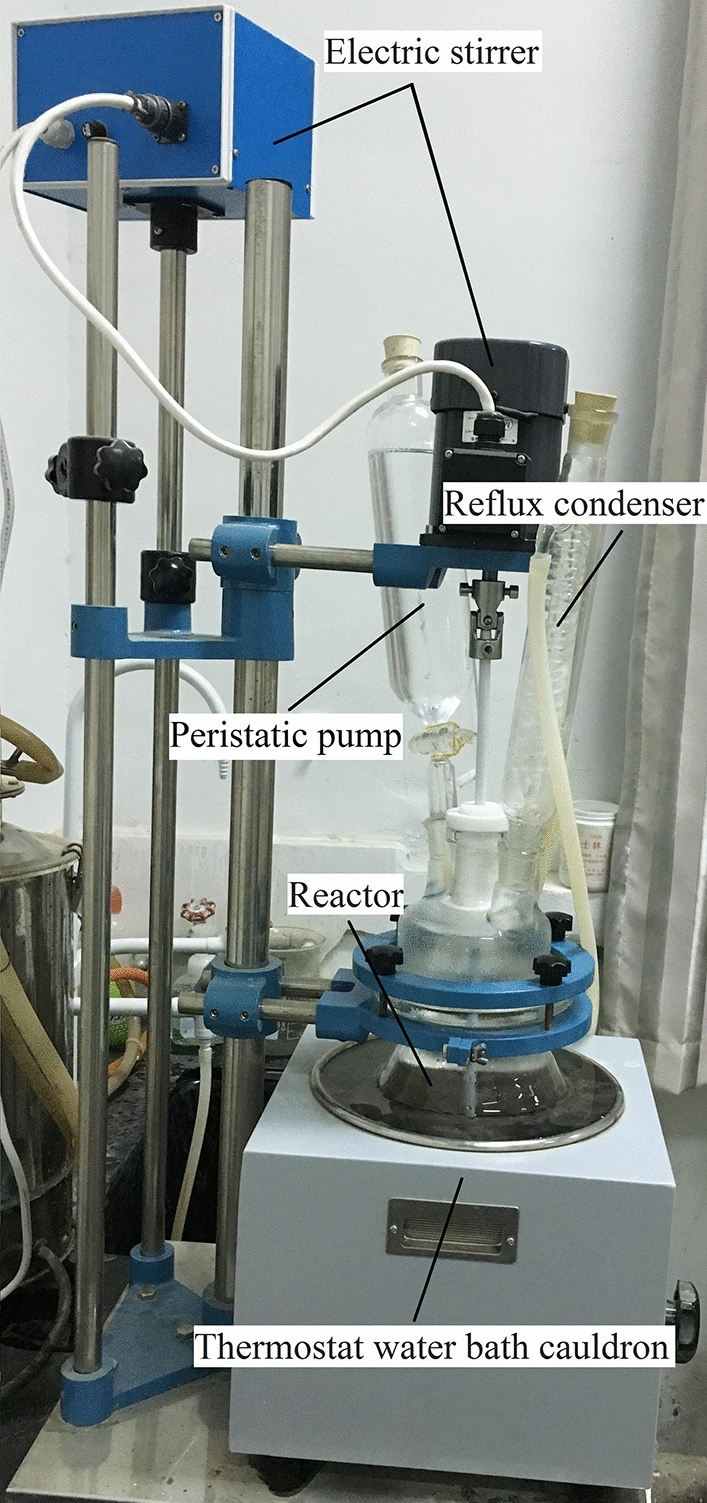
Setup for preparation of the PHA solution.

As shown in Fig. 1, first, 0.5 mol/L AlCl_3_ solution was added into a 2 L glass reactor and then heated to 60 °C. Next, a given volume of NaOH solution with a concentration of 0.5 mol/L was added to the reactor dropwise at a speed of 50 mL/min with rapid agitation. The total Al concentration of the PHA solution was 0.1 mol/L, and the OH/Al molar ratio was controlled at 2.5. Finally, the PHA solution was allowed to rest for 1 week at room temperature before ^27^Al NMR analysis, and the content of Al_13_ reached 94.73%.

The expansive soil selected for this study was sampled in Nanning, Guangxi Province, China. The basic physical properties of expansive soil are listed in Table 1.

**Table 1:** Basic physical properties of expansive soil.

Property	Test value
**Grain size distribution (%)**	
> 75 μm	3.90
2 μm–75 μm	35.70
< 2 μm	60.40
Specific gravity	2.70
**Atterberg limits (%)**	
Liquid limit	63.00
Plastic limit	24.94
Unified soil classification	CH
Clay mineral	Illite
LOI (%)	0.40
**Compaction properties (dry compaction method)**	
Optimum moisture content (%)	16.20
Std. proctor maximum dry unit weight, γ = ρg (kN/m^3^)	18.00
**Swell properties**	
Swell percent (%)	19.00
Swell pressure (kPa)	182.72

*LOI* loss of ignition.

Previous researchers have indicated that cation exchange is a key factor influencing clay-Al_13_ interactions. The cation exchange capacity mainly depends on the charge and hydrated radius of cations^32^, which is inversely proportional to the hydrated radius of cations and increases with the charge. Therefore, the ratio of charge to hydrated radius can be used to explain the cation selectivity of soil. As shown in Table 2, compiled from reference^33^, the charge-radius ratio of Al_13_ is higher than that of other cations, which indicates that the order of cation exchange capacity is Al_13_ > Ba^2+^ > Ca^2+^ > Mg^2+^ > K^+^ > Na^+^ > Li^+^. Therefore, in expansive soil stabilized with PHA solution, the cations will be replaced by Al_13_ ion groups, resulting in the modified effects.

**Table 2:** Charge-radius ratio of common cations and Al_13_.

Cation	Ionic radius (Å)	Hydrated radius (Å)	Charge-radius ratio
Li^+^	0.60	3.82	0.262
Na^+^	0.95	3.58	0.279
K^+^	1.33	3.31	0.302
Mg^2+^	0.65	4.28	0.467
Ca^2+^	0.99	4.12	0.485
Ba^2+^	1.35	4.04	0.495
Al_13_	–	9.50	0.737

Compiled from reference^33^.

### Sample preparation

To evaluate the influence of Al_13_ on the engineering properties of expansive soil, laboratory tests were conducted on natural and stabilized soil samples with various volumes of PHA solution. The Al/soil ratio (defined as the ratio of the amount of total Al in the PHA solution to the mass of expansive soil) was controlled at 0.10, 0.14, 0.18, 0.22, and 0.26 mmol/g.

According to the JTG E40-2007 (Chinese standard), expansive soil samples were ground and passed through a 0.5 mm sieve. Then, the soil particles were dried at 105 °C for 24 h in a drying oven. Dried expansive soil was exactly weighed and transferred to Bunsen beakers. Different volumes of PHA solution with a concentration of 0.1 mol/L were added with vigorous stirring. The clay suspensions were stored at 20 °C for 5 weeks, and then the supernatants were removed. The crosslinked expansive soil was oven dried and then ground and passed through a 0.5 mm or a 2 mm sieve to prepare the soil samples.

The removed supernatants of the above clay suspension were sampled for ^27^Al NMR analysis. The results indicated that Al_13_ or other Al monomers were not detected in the supernatants, which illustrated that all the Al_13_ groups and Al monomers were adsorbed by the expansive soil in the crosslinking process and replaced the low valence cations, which led to the modified effects.

### Testing procedures

#### Physio-mechanical property tests

The liquid limit (LL), plastic limit (PL) and grain size distribution of natural and stabilized soil samples were determined according to Chinese standard JTG E40-2007.

Furthermore, the standard JTG E40-2007 presents methods to measure the swelling potential of expansive soil, which includes the swell percent and swell pressure. The swell percent and swell pressure tests were both conducted on samples with diameters of 61.8 mm and heights of 20 mm. Each sample at the optimum moisture content and the maximum dry density was statically compacted into a traditional oedometer ring and then inundated with water. Samples for the swell percent test could swell freely, and the readings of the dial gauge were periodically recorded at pre-determined times. Other swell pressure tests were completely restricted to no height variation under vertical pressure. This pressure value was recorded until it reached a steady state.

Unconsolidated-undrained (UU) triaxial tests were conducted on the saturated soil samples based on JTG E40-2007. The samples were moulded into thin stainless steel tubes 76 mm in height and 38 mm in diameter at the optimum moisture content and maximum dry density. Then, the stress–strain curves could be obtained under a confining pressure of 100 kPa.

#### XRD and SEM analysis

X-ray diffraction (XRD) was conducted on a D8 Advance X-ray diffractometer (Cu Kα source) at a tube voltage of 40 kV and current of 40 mA. The homogeneous powder (< 20 μm) for the XRD test was ground with an agate mortar at 20 °C and 60% relative humidity. For the scanning electron microscopy (SEM) test, the microstructures of all soil samples (gold-coated) were examined under an FEI Quanta 250 at 20 kV.

## Results and discussion

### Atterberg limits

**Figure 2:**
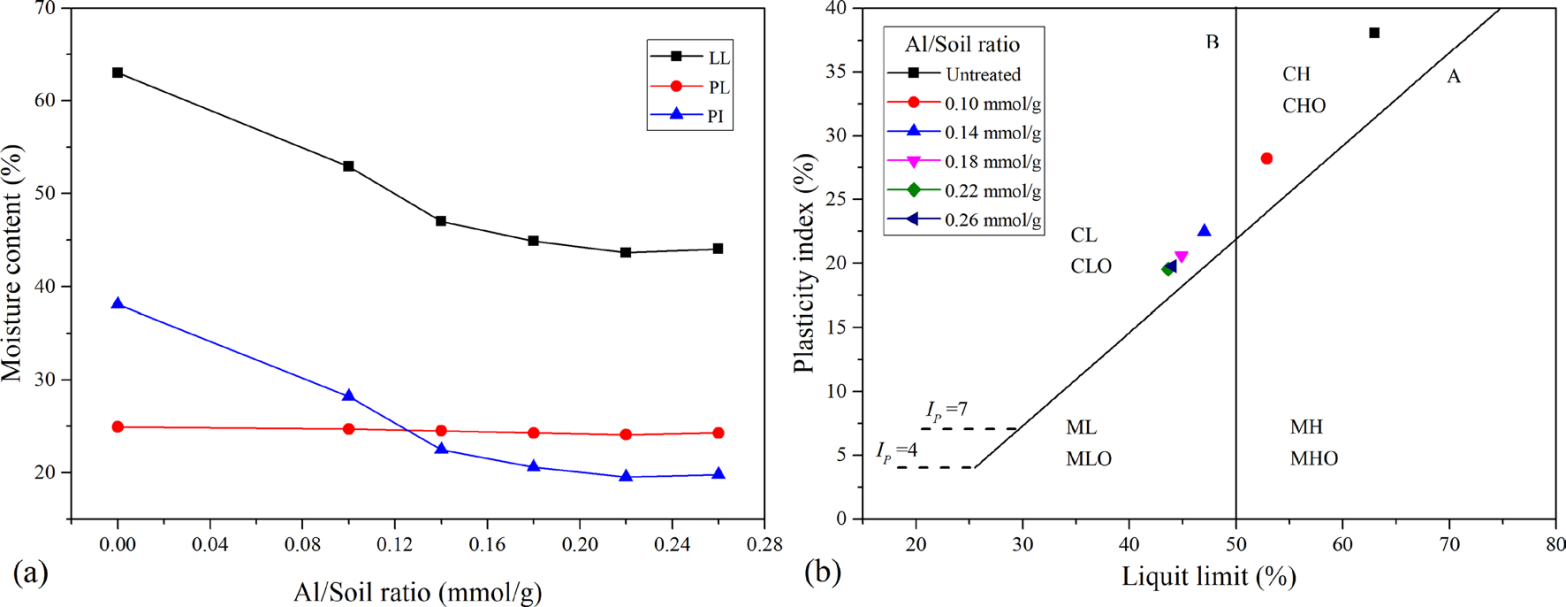
Atterberg limits and plasticity chart of stabilized expansive soil with a variable Al/soil ratio: (**a**) Atterberg limits; (**b**) plasticity chart.

The effects of Al_13_ on the liquid limit, plastic limit and plasticity index of expansive soil are presented in Fig. 2.

The liquid limit and plasticity index of the samples decreased as the Al/soil ratio increased, whereas the plastic limit decreased slightly. The minimum values of the liquid limit and plasticity index were 43.64% and 19.55, respectively, at the Al/soil ratio of 0.22 mmol/g, and then reached relatively stable values of 44.05% and 19.78. The decrease in the plasticity index indicated that the water stability of expansive soil improved and its water-holding capacity was reduced. This phenomenon can be explained by the marked reduction in the diffuse double layer (DDL) thickness of clay particles due to cation exchange between the expansive soil and Al_13_. On the other hand, because of the hydroxyl groups and large positive charge, Al_13_ could adsorb some water molecules, so the plastic limit of the soil treated with Al_13_ did not change significantly. Furthermore, Fig. 2b shows that expansive soil changed from a high-liquid-limit clay (CH) to a low-liquid-limit clay (CL), except for in the 0.10 sample. This observation implies that the grain size distribution of expansive soil changed.

### Grain size distribution

**Figure 3:**
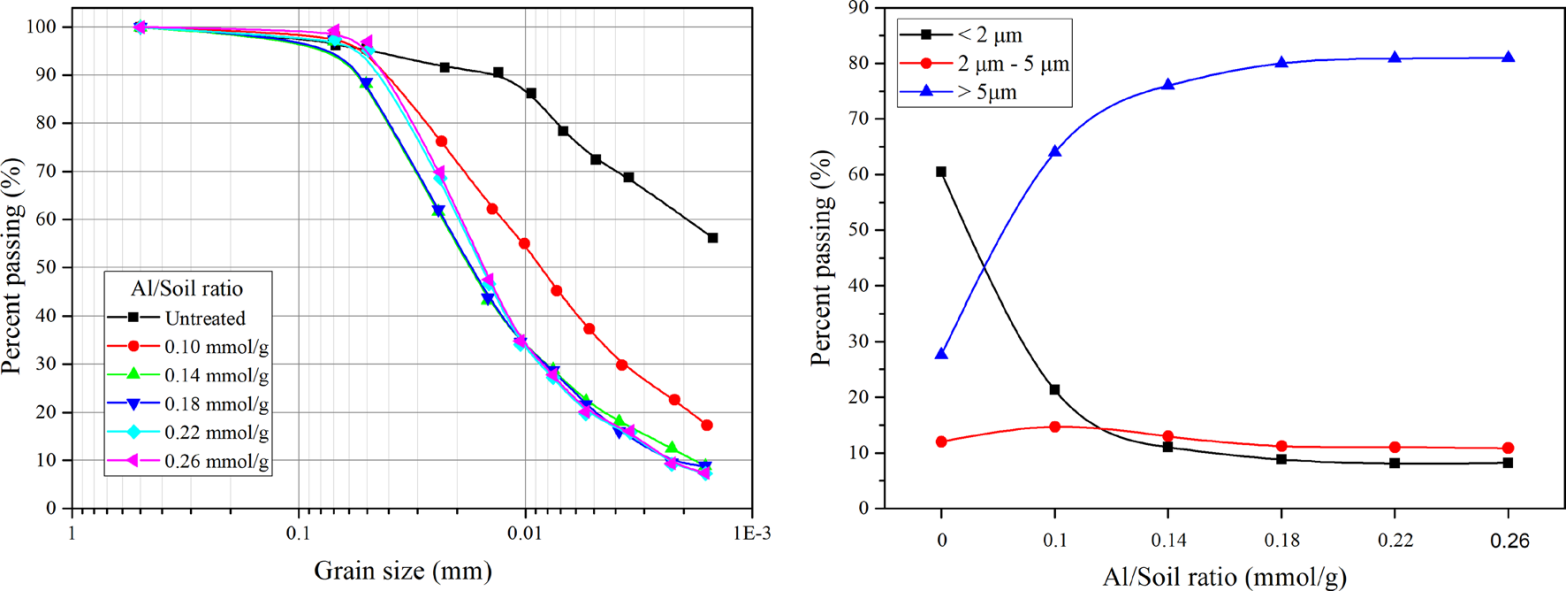
Grain size distribution of stabilized expansive soil with variable Al/soil ratios.

In accordance with the abovementioned standard, grain size distribution tests were performed on the stabilized soil samples with a variable Al/soil ratio. The test results obtained are presented in Fig. 3.

Table 1 shows that the clay and silt contents of natural expansive soil were 60.4% and 35.7%, respectively. However, adding PHA solution to the expansive soil reduced the clay content and increased the silt content, which can be explained by the interaction between expansive soil and Al_13_. The Al_13_ groups were absorbed onto the surface of the soil particles and then intercalated into the crystal layer to form a relatively stable structure. As a result, the low-valent cations in the soil particles were gradually replaced by Al_13_, and the thickness of the diffuse double layer (DDL) decreased. Therefore, the crystalline swelling and osmotic swelling stages of expansive soil were inhibited, resulting in a decrease in the clay dispersivity and an improvement in the stability of soil aggregates. Furthermore, as the Al/soil ratio increased beyond 0.18 mmol/g, the grain size distribution of stabilized soil remained nearly constant. This finding may be explained by the limited adsorption capacity of the Al_13_ groups in the clay surfaces, which suggests that too much PHA solution will not have a better modification effect on expansive soil.

### Swell percent and swell pressure tests

**Figure 4:**
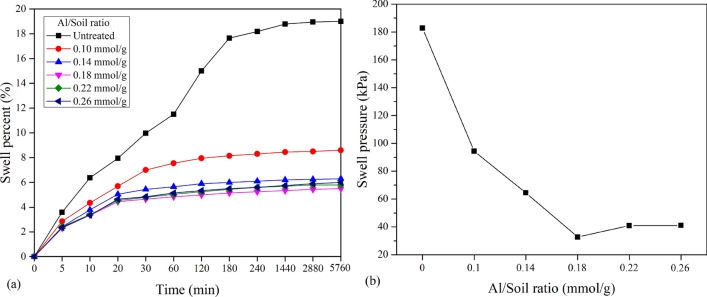
Swell percent and swell pressure of stabilized expansive soil with a variable Al/soil ratio: (**a**) swell percent; (**b**) swell pressure.

The swell percent and swell pressure of expansive soil with variable Al/soil ratios are given in Fig. 4. The results show that the swell percent and swell pressure of all soil samples decreased with an increase in the Al/soil ratio.

As shown in Fig. 4a, the swell percent increased greatly in the initial stage of the experiment and then became steady over time. The Al/soil ratios of 0.18 and 0.26 mmol/g reduced the swell percent from the initial value of 19% to 5.5% and 6%, respectively. Moreover, the swell pressure presented a significant decrease from the initial value of 182.72 kPa to 32.74 kPa at an Al/soil ratio of 0.18 mmol/g. When the Al/soil ratio exceeded 0.10 mmol/g, the stabilized soil almost changed to non-swelling soil. This phenomenon can be attributed to cation exchange between Al_13_ and the expansive soil. The replaceable cations of expansive soil were substituted by Al_13_, which enhanced the flocculation of soil particles and reduced the specific surface area and water affinity, which ultimately decreased the swelling potential^34^. Hence, a certain amount of PHA solution will cause a decrease in swell percent and repulsive forces between clay particles. Furthermore, it is noteworthy that the lowest swell percent and swell pressure were observed when the Al/soil ratio was 0.18 mmol/g rather than 0.26 mmol/g. The reason for this phenomenon is that the excessive Al_13_ ion groups disrupt the equilibrium of the adsorption system because of inhomogeneous charges on the clay surface^19^. As a result, when the Al/soil ratio continued to increase, the swell percent and swell pressure presented a slight increase followed by a stable state after achievement of a new equilibrium.

### Unconsolidated-undrained (UU) test

**Figure 5:**
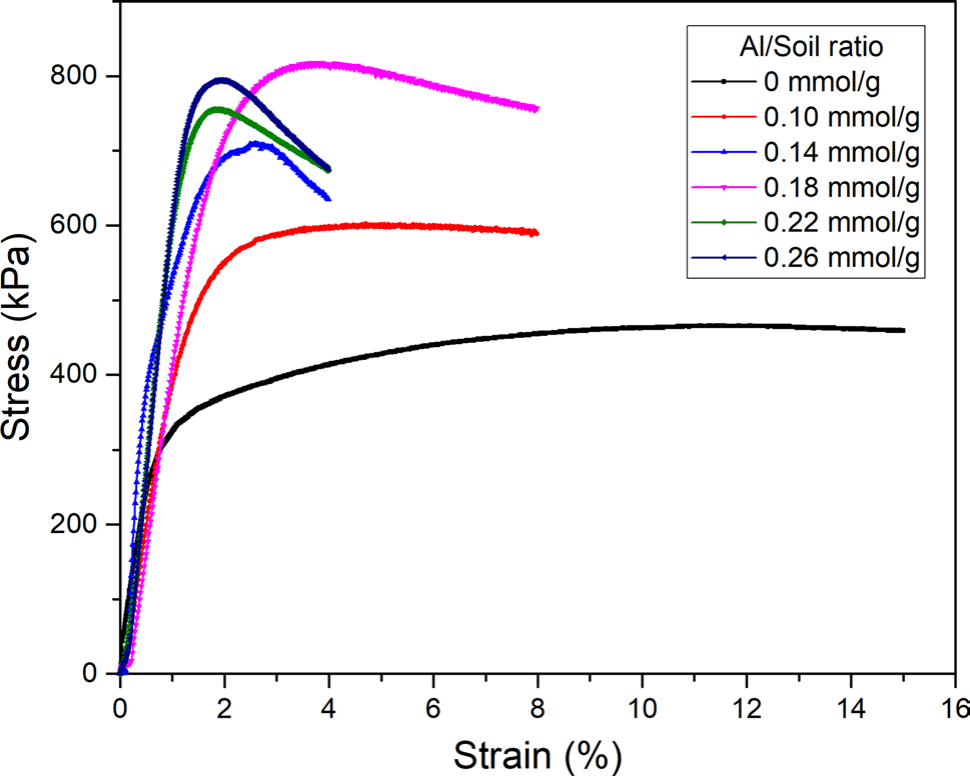
Stress versus strain of stabilized expansive soil with a variable Al/soil ratio.

The effect of Al_13_ on the stress–strain behaviour of stabilized soil under an effective confined pressure of 100 kPa is shown in Fig. 5. It is generally recognized that the stress–strain curve can be divided into two types: strain hardening and strain softening. As shown in Fig. 5, the stress–strain curve was transformed from the strain hardening type to the strain softening type as the Al/soil ratio increased. This phenomenon indicates that the structure and arrangement of the soil particles changed.

Furthermore, it can be observed that the unconsolidated-undrained shear strength of expansive soil was enhanced by Al_13_, significantly increasing from 0.46 to 0.82 MPa with Al/soil ratios ranging from 0 to 0.18 mmol/g. This increase may be attributed to the flocculation and agglomeration of clay particles caused by cation exchange in the soil-Al_13_ solution. When the Al/soil ratio continued to increase, the shear strength tended to decrease from 0.82 to 0.75 and 0.78 MPa, respectively. This phenomenon can be due to the explanation presented in Sect. 3.3. When excessive Al_13_ ion groups were added to the soil samples, the balance between the Al_13_ groups and negative ions in the clay particles would be disrupted due to the inhomogeneous charges on the clay surface, which destabilized the intercalation and adsorption system and ultimately affected the process of flocculation–agglomeration of clay particles, resulting in a reduction in shear strength. However, the shear strength presented an increase as the Al/soil ratio increased from 0.22 to 0.26 mmol/g. This observation is attributed to the conversion of excessive Al_13_ into the other Al species, such as monomeric Al and Al_n_(OH)_3n_ or Al_2_O_3_, which enhance the cementation for improving the cohesive force of clay aggregates, resulting in an increase in shear strength^19^.

### XRD and SEM analyses

**Figure 6:**
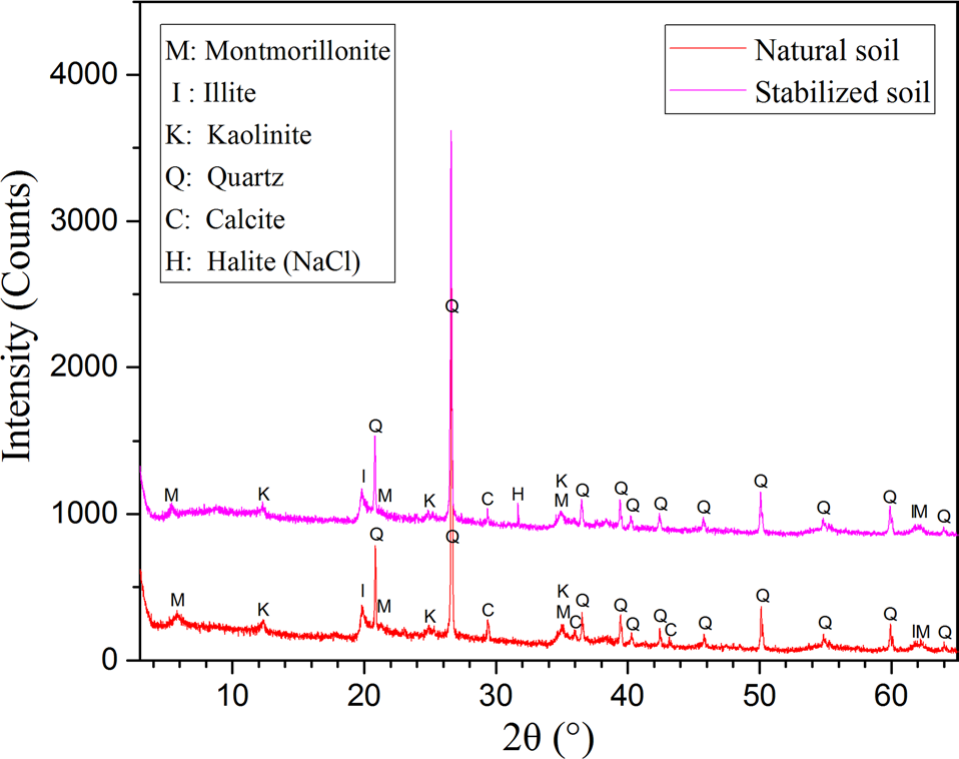
XRD patterns of natural soil and stabilized soil with an Al/soil ratio of 0.18 mmol/g.

**Figure 7:**
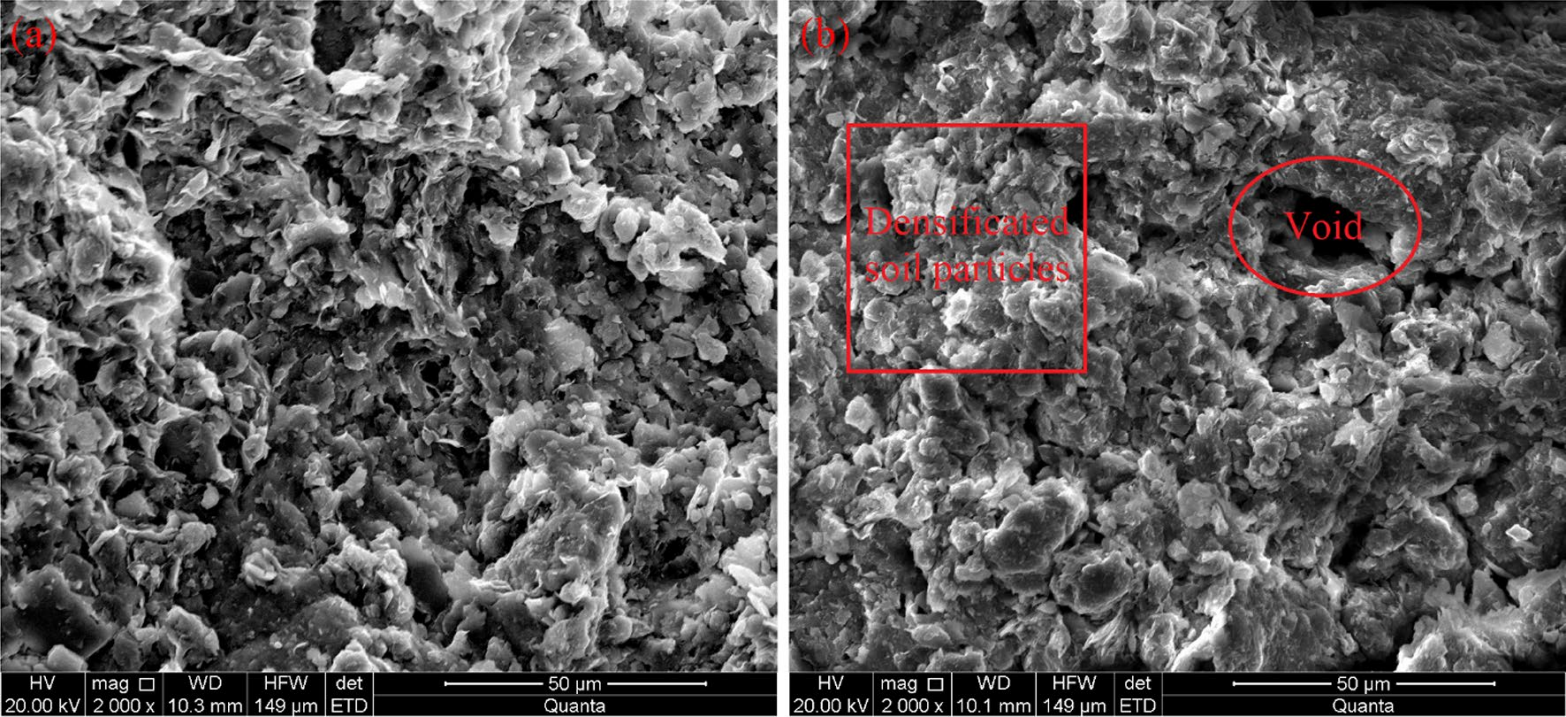
SEM images of soil samples: (**a**) natural expansive soil; (**b**) stabilized soil with an Al/soil ratio of 0.18 mmol/g.

As a result of the above analyses, the optimum Al/soil ratio for treating expansive soil was determined to be 0.18 mmol/g when combining the plasticity index, clay content, swelling potential and unconsolidated-undrained shear strength. Therefore, XRD and SEM analyses were performed on natural and stabilized soil samples with an Al/soil ratio of 0.18 mmol/g. The XRD and SEM images are shown in Figs. 6 and 7.

As shown in Fig. 6, the main mineral compositions of the natural and stabilized soil samples were quartz, montmorillonite, illite and calcite. Almost the same mineral compositions were found in both soil samples, except a small amount of halite was found in the diffraction pattern of the stabilized expansive soil. This may be attributed to the crystallization of sodium chloride during soil drying, which was derived from the PHA solution and expansive soil.

It can be seen from Fig. 7a that natural expansive soil shows a laminar structure with large and thin clay flakes, which is the typical soil morphology of clay minerals, and is in agreement with Goodarzi et al.^35^ and Keller^36^. With the addition of PHA solution, the clay particles transformed from a dispersive structure to a flocculated-agglomerated structure featuring the densification of particle associations accompanied by distinct voids. The hydrated Al_13_ ion groups intercalated into the crystal layer to form a relatively stable interlayer structure. Meanwhile, these Al_13_ ion groups were also adsorbed onto the surface of the clay particles to increase the cementation for promoting clay aggregates to become compactly bound with other clay particles. The resultant agglomeration reduced the surface area and inhibited the crystalline swelling and osmotic swelling of expansive soil, which in turn improved the geotechnical properties.

### Discussion

Based on the above analyses, Al_13_ can greatly reduce the swelling potential of expansive soil and greatly improve its physio-mechanical properties. However, it is generally believed that the element Al in PHA solution will produce aluminium toxicity and harm the growth of animals and plants^37,38^. However, the majority of Al_13_ groups will enter the interlayer of montmorillonite crystals and will be adsorbed and stabilized considerably on the surfaces of negatively charged clay particles^39,40^, consequently forming stable complexes that are strongly fixed in the solid phase of soils and seldom released^41^. Additionally, excessive Al_13_ in the soil solution will decompose due to reactions with humic acid and other organic matter^42^. In this respect, Syuntaro^41^ indicated that Al_13_ could not be a major plant growth inhibitor in humus-rich or negatively charged soils (such as expansive soil). In fact, hydroxy-aluminium is widely used as a flocculant in water and wastewater treatment^43^. Therefore, the adverse effects of PHA solution on the environment will be greatly reduced or even eliminated. Among them, the effects of the PHA solution on the carbon emissions of soil needs further study. Moreover, it should be emphasized that treatment of expansive soil with Al_13_ is utilized to meet engineering requirements and the stabilized soil is generally not used for the growth and cultivation of crops, so the impact of Al_13_ on soil organic matter is not a key point.

In practical engineering, the durability of stabilized soil is a common problem of many traditional stabilizers^44–49^. However, Yazdandoust et al.^50^ claimed that the beneficial effects of polymer stabilization is preserved and not lost after cyclic wetting and drying. As one of the polymers, Al_13_ is the primary component of intercalated Al adsorbed in the crystal layer and has good thermal stability after crosslinking with montmorillonite. As a result, the treatment of expansive soil by Al_13_ shows higher potential durability than traditional stabilizers. In addition, we tried to optimize the synthetic parameters to achieve large-scale preparation of PHA solutions with a high and stable content of Al_13_, and some technical means were prepared to achieve the purification, concentration and powdering of PHA solution to facilitate its transportation and storage. Moreover, we planned to make the PHA solution infiltrate into expansive soil relatively uniformly by model tests and field tests, combining physical means such as borehole filling, surface spraying and electroosmosis, electrophoresis and other electrochemical methods. Currently, we are conducting model tests by means of electroosmosis and surface spraying. The results show that these two methods are practicable and effective. Therefore, the treatment of expansive soil by Al_13_ may be an effective and promising engineering method.

## Conclusions

In this study, the physio-mechanical characteristics of expansive soil stabilized with Al_13_, such as Atterberg limits, grain size distribution, swelling potential and shear strength, were investigated. The influence of Al_13_ on the micro-morphology of expansive soil was also examined. The following conclusions of this study can be summarized from the test results:

1. The physio-mechanical properties of expansive soil significantly improved after treatment with PHA solution. At an Al/soil ratio of 0.18 mmol/g, the plasticity index and clay content of stabilized soil decreased from 38.06 to 20.6 and from 60.4% to 8.8%, respectively. Moreover, the swell percent and swell pressure presented a significant decrease from the initial values of 19% to 5.5% and 182.72 kPa to 32.74 kPa, respectively. The unconsolidated-undrained shear strength of expansive soil was enhanced by Al_13_, significantly increasing from 0.46 to 0.82 MPa.
2. Stabilized expansive soil presented a remarkable densification of soil aggregates compared to the microstructure observed for natural expansive soil. This phenomenon can be explained by the flocculation–agglomeration of soil particles due to the cation exchange and intercalated adsorption of Al_13_.
3. The consistency of the enhancement in physio-mechanical properties and the micro-structural changes of stabilized expansive soil indicated that Al_13_ is a suitable stabilizing material for expansive soil. Thus, the content of Al_13_ in PHA solution is a key factor influencing the treatment of expansive soil.
4. The optimum Al/soil ratio for expansive soil stabilized with PHA solution is 0.18 mmol/g under comprehensive consideration of the improvement in properties. This value mainly depends on the properties of the expansive soil as well as the Al_13_ content of the PHA solution.
